# A Latent Autoantibody Axis Associated with Vascular Vulnerability in Ischemic Stroke: Integrated Statistical and Machine-Learning Analysis

**DOI:** 10.3390/ijms27052465

**Published:** 2026-03-07

**Authors:** Tomohiro Sugiyama, Yoichi Yoshida, Takaki Hiwasa, Masaaki Kubota, Seiichiro Mine, Yoshinori Higuchi

**Affiliations:** 1 Department of Neurological Surgery, Graduate School of Medicine, Chiba University, Chiba 260-8670, Japan; 2 Department of Neurosurgery, Chiba Kaihin Municipal Hospital, Chiba 261-0012, Japan; 3 Department of Neurosurgery, Chiba Prefectural Sawara Hospital, Chiba 287-0003, Japan; 4 Department of Neurosurgery, Gyotoku General Hospital, Chiba 272-0103, Japan

**Keywords:** ischemic stroke, autoantibodies, vascular vulnerability, biomarker integration, machine learning, random forest, principal component analysis, immune activation, risk stratification, precision medicine

## Abstract

Ischemic stroke remains a major cause of mortality and long-term disability worldwide, and improved strategies for identifying individuals at elevated vascular risk are needed. Serum autoantibodies have emerged as potential biomarkers reflecting vascular injury and immune activation; however, their integrative biological significance and incremental predictive value beyond established clinical risk factors remain unclear. We analyzed 833 participants, including patients with acute ischemic stroke (AIS) or transient ischemic attack (TIA) and healthy controls. Serum levels of anti-PDCD11 antibody (Ab), anti-DNAJC2 antibody, and anti-PAI-1 (SERPINE1) antibody were quantified, and multivariable logistic regression and machine-learning (ML) models (logistic regression and random forest) were constructed using clinical variables with and without antibody markers. Model performance was evaluated using cross-validation, bootstrap-derived confidence intervals, calibration metrics, and reclassification indices. Model interpretability analyses, principal component analysis (PCA), unsupervised clustering, and propensity score matching were performed to explore latent biological structures. Clinical-only models demonstrated excellent discrimination (bootstrap Area Under the Curve (AUC) 0.917 for random forest and 0.919 for logistic regression). The addition of antibody markers yielded similar performance (AUC 0.913 and 0.923, respectively) without evidence of meaningful improvement in reclassification. However, SHapley Additive exPlanations (SHAP) analysis identified antibody markers as influential contributors following major clinical risk factors. PCA revealed a dominant antibody component explaining approximately 79% of the variance, which remained independently associated with stroke after age adjustment. Unsupervised clustering further identified a high-risk subgroup characterized by consistently elevated antibody levels. These findings support the presence of a latent antibody axis associated with vascular vulnerability. Although antibody markers did not substantially enhance global predictive performance, they captured integrated biological signals reflecting cumulative vascular and immunological stress. Autoantibody profiling may complement conventional risk assessment by improving biological characterization of stroke susceptibility. Prospective validation in independent cohorts is required prior to clinical implementation.

## 1. Introduction

Ischemic stroke remains a leading global cause of mortality and long-term disability [1]. Although conventional vascular risk factors—including hypertension, diabetes mellitus, dyslipidemia, and cardiovascular disease—are well established, they do not fully explain individual susceptibility to stroke, particularly among younger patients or those without prominent clinical risk profiles [2]. Biomarkers capable of capturing latent vascular injury and subclinical pathophysiological processes may therefore enhance risk characterization and provide biological insight beyond traditional clinical variables [3]. Notably, a subset of patients—including younger individuals and those lacking overt conventional risk factors—remains difficult to identify using traditional risk models. This clinical gap has driven increasing interest in biomarker-based approaches that may capture orthogonal dimensions of vascular risk beyond established clinical predictors.

Growing evidence suggests that serum autoantibodies reflect underlying vascular damage, inflammatory activation, and endothelial dysfunction preceding clinically overt disease [4]. Rather than functioning as isolated indicators, these immune responses may represent integrated biological signals associated with vascular vulnerability [5]. Importantly, such signals may reflect cumulative biological stress and shared immunological pathways rather than discrete pathological events.

Among the growing number of candidate autoantibody markers, antibodies targeting DNAJC2, PDCD11, and SERPINE1 have been reported to be elevated in patients with ischemic stroke and related vascular conditions [6,7,8]. These proteins represent biologically distinct yet complementary pathways, including cellular stress responses (DNAJC2), apoptosis-related signaling (PDCD11), and fibrinolytic regulation (SERPINE1). Together, these processes suggest that combined evaluation of these antibodies may provide a more comprehensive representation of stroke-related pathophysiology than any single marker alone and may reveal latent biological structures underlying vascular vulnerability.

Prior work has indicated that single biomarkers often provide limited diagnostic utility, whereas multi-marker approaches may better capture the complex and interconnected pathways involved in stroke pathogenesis [3,9]. This perspective aligns with contemporary biomarker research recognizing that apoptosis, inflammation, endothelial stress, and thrombotic mechanisms interact in nonlinear and multidimensional ways [5]. However, whether such antibody markers reflect independent effects or represent a shared underlying biological axis remains unclear.

Machine-learning (ML) methods offer a flexible framework for modeling such biological complexity. In particular, tree-based algorithms can detect nonlinear relationships and higher-order interactions that may not be adequately captured by traditional regression models [10]. Beyond improving predictive performance, ML approaches may also help reveal latent biological structures within biomarker data and characterize heterogeneous risk phenotypes [11].

Accordingly, the present study investigated the integration of anti-PDCD11 antibody, anti-DNAJC2 antibody, and anti-PAI-1 (SERPINE1) antibody levels with clinical variables to enhance risk characterization for ischemic stroke. In addition to evaluating predictive performance, we sought to determine whether combined antibody profiles reflect a shared biological signal associated with stroke vulnerability and whether such patterns may define distinct immunologically informed risk phenotypes.

## 2. Results

### 2.1. Clinical Characteristics

A total of 543 patients with ischemic stroke (457 acute ischemic stroke (AIS) and 86 transient ischemic attack [TIA]) and 284 healthy controls were included in the analysis. Patients with ischemic stroke were significantly older than controls and exhibited higher prevalences of hypertension, diabetes mellitus, dyslipidemia, and cardiovascular disease (Table 1).

### 2.2. Serum Antibody Levels and Diagnostic Performance

**Figure 1:**
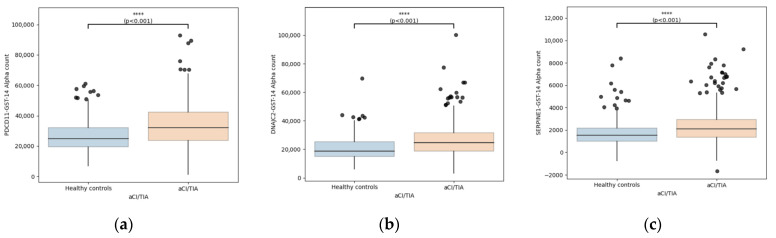
Distribution of serum autoantibody levels in patients with ischemic stroke and healthy controls. Boxplots illustrate GST-corrected AlphaLISA signal intensities for PDCD11-Ab (**a**), DNAJC2-Ab (**b**), and SERPINE1-Ab (**c**) in healthy controls and patients with acute ischemic stroke or transient ischemic attack (AIS/TIA). Boxes represent the interquartile range (IQR), horizontal lines indicate medians, whiskers extend to 1.5 × IQR, and dots represent individual data points. Comparisons between groups were performed using the Mann–Whitney U test. **** *p* < 0.001.

All three antibody markers—PDCD11-Ab, DNAJC2-Ab, and SERPINE1-Ab—were significantly elevated in patients with ischemic stroke compared with controls (Figure 1).

**Figure 2:**
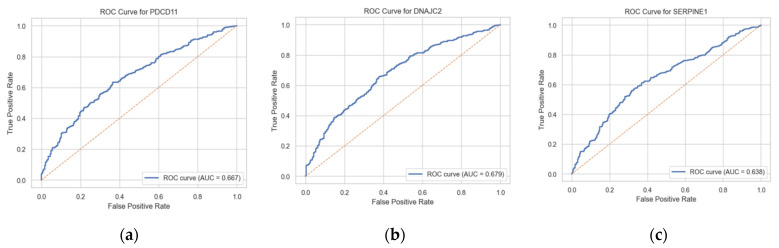
Receiver operating characteristic (ROC) curves for individual serum autoantibody markers in the identification of ischemic stroke. ROC curves are shown for PDCD11-Ab (**a**), DNAJC2-Ab (**b**), and SERPINE1-Ab (**c**). The area under the curve (AUC) values were 0.667 (95% CI 0.629–0.704) for PDCD11, 0.679 (95% CI 0.641–0.715) for DNAJC2, and 0.638 (95% CI 0.600–0.679) for SERPINE1. The dashed diagonal line represents the line of no discrimination (AUC = 0.5). These findings indicate modest discriminative performance of individual antibody markers for ischemic stroke.

Receiver operating characteristic (ROC) analysis demonstrated modest discriminative ability for each individual marker, with AUC values of 0.67 (95% CI 0.629–0.704) for PDCD11-Ab, 0.68 (95% CI 0.641–0.715) for DNAJC2-Ab, and 0.64 (95% CI 0.600–0.679) for SERPINE1-Ab (Figure 2).

Although the diagnostic performance of single antibody markers was limited, these findings suggest that each marker may reflect stroke-related biological processes, even though their standalone discriminative performance was modest.

### 2.3. Multivariable Logistic Regression Analysis

Multivariable logistic regression was performed to evaluate independent associations between clinical variables, antibody markers, and ischemic stroke (Table 2).

In multivariable analysis, age, hypertension, and diabetes mellitus were significantly associated with stroke. Among the antibody markers, DNAJC2-Ab remained independently associated, whereas PDCD11-Ab and SERPINE1-Ab were not statistically significant after adjustment.

The McFadden pseudo-R^2^ was 0.51, and the likelihood ratio test indicated overall model significance (*p* < 0.001).

### 2.4. Machine-Learning Model Performance

Machine-learning models were evaluated using cross-validation within the training dataset and subsequently assessed on the independent test dataset using bootstrap resampling to estimate 95% confidence intervals.

Using clinical variables alone, the Random Forest model achieved a bootstrap mean AUC of 0.917 (95% CI 0.876–0.950), while logistic regression yielded a bootstrap mean AUC of 0.919 (95% CI 0.879–0.953).

**Figure 3:**
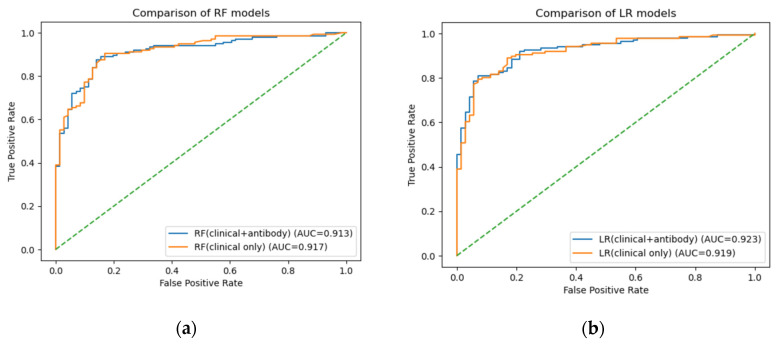
Receiver operating characteristic (ROC) curves comparing machine-learning models with and without antibody markers for the prediction of ischemic stroke. (**a**) Random Forest (RF) models. (**b**) Logistic Regression (LR) models. Blue curves represent models including clinical variables and antibody markers, whereas orange curves represent models using clinical variables only. The area under the curve (AUC) values for the test dataset were 0.913 (RF with antibodies) and 0.917 (RF clinical only), and 0.923 (LR with antibodies) and 0.919 (LR clinical only), respectively. The dashed diagonal line indicates no discrimination (AUC = 0.5). These findings demonstrate that the addition of antibody markers did not materially improve overall discriminative performance.

After incorporating antibody markers, the Random Forest model demonstrated a bootstrap mean AUC of 0.913 (95% CI 0.873–0.948), and logistic regression achieved a bootstrap mean AUC of 0.923 (95% CI 0.884–0.954) (Figure 3).

**Figure 4:**
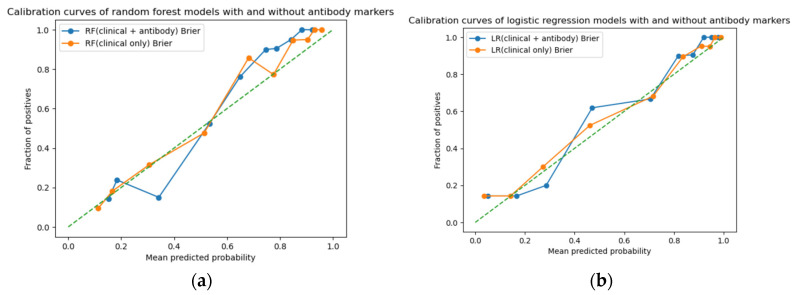
Calibration plots of machine-learning models with and without antibody markers for the prediction of ischemic stroke. (**a**) Random Forest (RF) models. (**b**) Logistic Regression (LR) models. Blue curves represent models including clinical variables and antibody markers, and orange curves represent clinical-only models. Points indicate observed event fractions within grouped predicted-probability bins, and the dashed diagonal line represents perfect calibration. Brier scores were 0.119 (clinical + antibody) and 0.112 (clinical only) for the Random Forest model, and 0.106 and 0.107, respectively, for logistic regression. These findings indicate preserved calibration and no material improvement in overall calibration performance with the addition of antibody markers.

Calibration analysis showed Brier scores of 0.119 and 0.112 for the Random Forest models with and without antibody markers, respectively, and 0.106 and 0.107 for the corresponding logistic regression models (Figure 4).

Reclassification metrics (NRI and IDI) did not indicate improvement with the addition of antibody markers and were numerically negative (Table 3).

### 2.5. Model Interpretability Using SHAP

**Figure 5:**
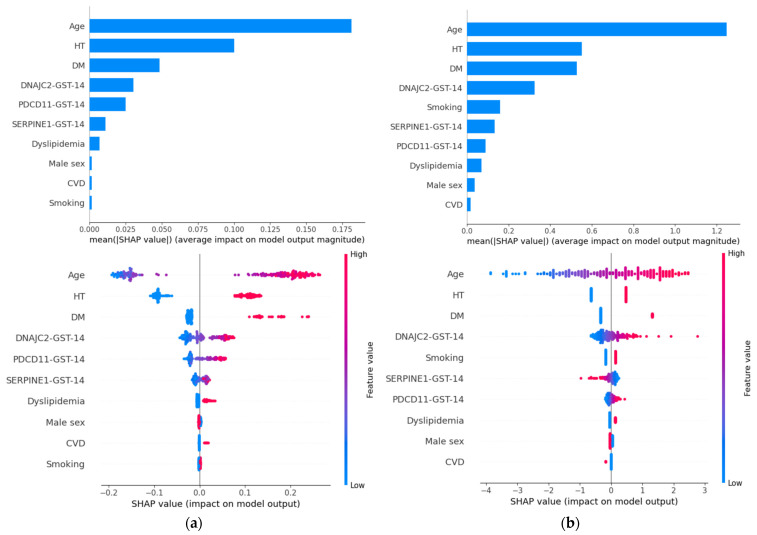
SHAP-based feature importance and summary plots for the (**a**) Random Forest and (**b**) Logistic Regression models. The upper panels display the mean absolute SHAP values, representing the average impact of each variable on model output magnitude. Age, hypertension (HT), and diabetes mellitus (DM) were the most influential predictors in both models. The three antibody markers (DNAJC2-Ab, PDCD11-Ab, and SERPINE1-Ab) ranked immediately after these established clinical risk factors. The lower panels show SHAP summary plots, where each point represents an individual sample. The horizontal axis indicates the SHAP value (impact on model output), and color represents the feature value (red: high; blue: low). Antibody markers demonstrated heterogeneous contributions across individuals, supporting their context-dependent effects within the prediction models.

SHAP analysis was performed to evaluate feature contributions and enhance model interpretability (Figure 5). In both the Random Forest and logistic regression models, age, hypertension, and diabetes mellitus emerged as the most influential predictors, consistent with established vascular risk factors.

Importantly, the three antibody markers—DNAJC2-Ab, PDCD11-Ab, and SERPINE1-Ab—ranked immediately after these clinical variables in terms of mean absolute SHAP values. Although the addition of antibody markers yielded limited improvement in global discrimination metrics, they contributed meaningfully to individual risk estimation within the models.

**Figure 6:**
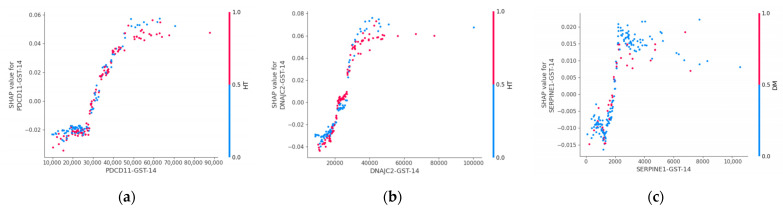
SHAP dependence plots illustrating nonlinear effects of antibody markers in the Random Forest model. The plots display the relationship between antibody levels (*x*-axis) and their corresponding SHAP values (*y*-axis), representing the contribution of each marker to stroke prediction. (**a**) Dependence plot for **PDCD11-Ab**, showing a nonlinear increase in SHAP values with increasing antibody levels, suggesting a threshold-like effect on predicted stroke risk. (**b**) Dependence plot for **DNAJC2-Ab**, demonstrating a similar nonlinear relationship between antibody levels and model output. (**c**) Dependence plot for **SERPINE1-Ab**, showing heterogeneous contributions across individuals, with evidence of interaction effects. In each panel, the x-axis represents antibody levels and the y-axis represents SHAP values, indicating the contribution of each marker to stroke prediction. Point color indicates interacting clinical variables (hypertension for PDCD11-Ab and DNAJC2-Ab; diabetes mellitus for SERPINE1-Ab), highlighting context-dependent effects of antibody levels on model predictions.

Dependence plots revealed nonlinear relationships between antibody levels and predicted stroke risk (Figure 6). Increasing levels of DNAJC2 antibody and PDCD11 antibody were associated with progressively higher SHAP values, whereas SERPINE1 antibody demonstrated greater heterogeneity across individuals. These patterns indicate that antibody markers may influence risk in a threshold- or context-dependent manner rather than through simple linear effects.

**Figure 7:**
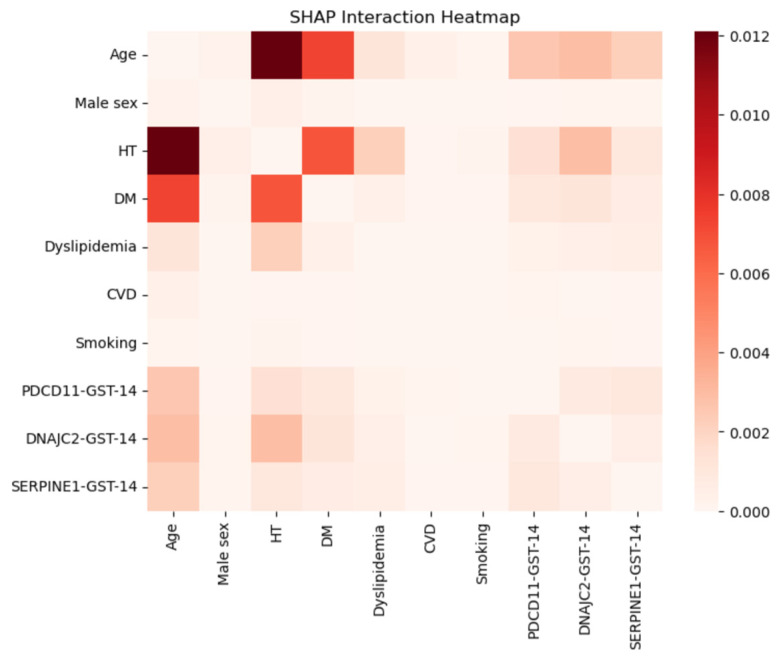
SHAP interaction heatmap derived from the Random Forest model. The heatmap illustrates pairwise interaction strengths between variables based on SHAP interaction values, where darker colors indicate stronger interactions contributing to model predictions. Age showed the strongest interaction with hypertension and diabetes mellitus, highlighting the dominant role of traditional vascular risk factors. Notably, antibody markers demonstrated measurable interactions with clinical variables, particularly with age and hypertension, suggesting that their effects on ischemic stroke risk may be context-dependent rather than purely additive. These findings further support the presence of nonlinear relationships captured by the Random Forest model.

SHAP interaction analysis further demonstrated that antibody markers primarily affected model predictions through interactions with established clinical risk factors, particularly age and hypertension (Figure 7). Together, these findings support the presence of complex, non-additive relationships captured by the machine-learning models.

### 2.6. Principal Component Analysis of Antibody Markers

**Figure 8:**
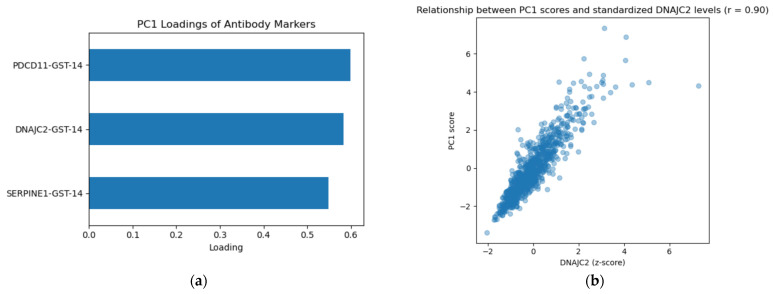
Principal component structure and clustering of antibody markers. (**a**) Loadings of individual antibody markers on the first principal component (PC1), demonstrating comparable contributions from PDCD11-Ab, DNAJC2-Ab, and SERPINE1-Ab. PC1 explained 79.3% of the total variance, indicating a strong shared antibody signal. (**b**) Relationship between PC1 scores and standardized DNAJC2-Ab levels (r = 0.899), supporting the interpretation that PC1 reflects overall antibody burden rather than a single-marker effect.

Principal component analysis (PCA) demonstrated a strong shared structure among the antibody markers (Figure 8). The first principal component (PC1) explained 79.3% of the total variance, while the first two components together accounted for approximately 93%, indicating that these markers represent a shared immunological signature rather than independent biological effects.

All three antibody markers contributed substantially and in a similar direction to PC1, suggesting coordinated elevation across individuals. PC1 scores were strongly correlated with standardized DNAJC2 antibody levels (r = 0.899), supporting the interpretation that this component reflects overall antibody burden rather than the influence of a single marker.

### 2.7. Association Between the Principal Antibody Component and Stroke

**Figure 9:**
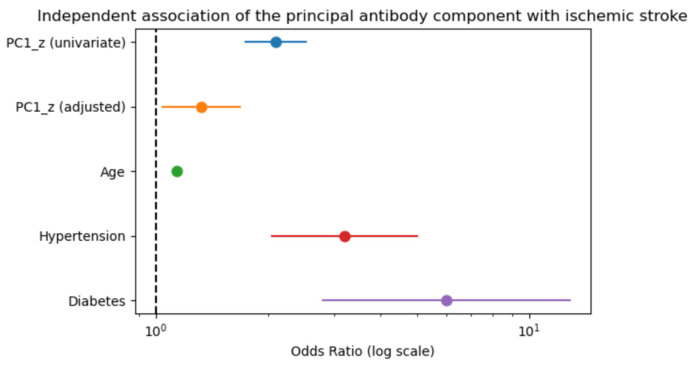
Independent association between the principal antibody component (PC1) and ischemic stroke. Forest plot showing odds ratios (ORs) with 95% confidence intervals derived from univariate and multivariable logistic regression analyses. PC1 was significantly associated with ischemic stroke in univariate analysis and remained independently associated after adjustment for age, hypertension, and diabetes mellitus.

The principal antibody component was significantly associated with ischemic stroke in univariate analysis (OR 1.62, 95% CI 1.43–1.82; *p* < 0.001). After adjustment for age, hypertension, and diabetes mellitus, PC1 remained independently associated with stroke (OR 1.25, 95% CI 1.08–1.45; *p* = 0.003) (Figure 9).

PC1 showed only a modest correlation with age (r = 0.30), suggesting that the antibody-related signal captures information beyond age-related vascular risk.

ROCanalysis demonstrated modest discrimination for PC1 alone (AUC = 0.679), indicating that while the composite antibody signal is independently associated with stroke, it is insufficient as a standalone predictive marker but may provide complementary biological insight when integrated with clinical risk factors.

### 2.8. Identification of Antibody-Defined Risk Phenotypes

**Figure 10:**
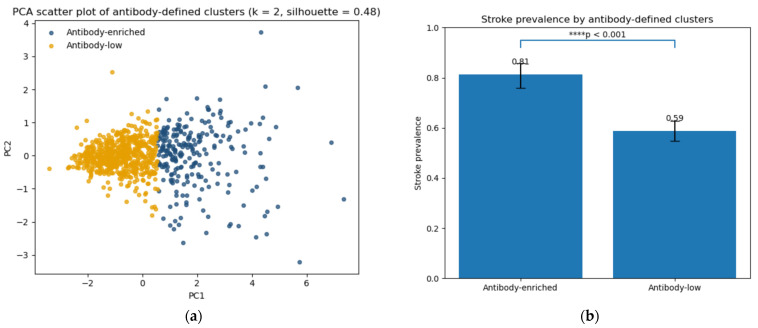
Antibody-defined risk phenotypes identified by unsupervised clustering. (**a**) Principal component analysis (PCA) scatter plot showing participant distribution based on antibody profiles. Colors indicate k-means clusters (k = 2; silhouette score = 0.48). (**b**) Stroke prevalence by cluster. The antibody-enriched cluster demonstrated significantly higher stroke prevalence compared with the antibody-low cluster (82% vs. 59%, χ^2^ *p* < 0.001). Statistical significance is indicated as follows: **** *p* < 0.001.

Unsupervised k-means clustering based on antibody markers identified two distinct subgroups with optimal separation (silhouette score = 0.48) (Figure 10). Visualization in principal component space demonstrated partial but meaningful separation between clusters.

One cluster exhibited substantially higher stroke prevalence than the other (82% vs. 59%, χ^2^ *p* < 0.001), indicating that antibody profiles may define biologically distinct risk phenotypes. Importantly, these findings suggest that the combined antibody pattern, rather than individual markers, may better capture heterogeneity in stroke risk.

This pattern-oriented approach may therefore provide a framework for identifying latent immunological risk phenotypes in ischemic stroke.

### 2.9. Sensitivity Analyses (See Appendix A)

**Figure A1:**
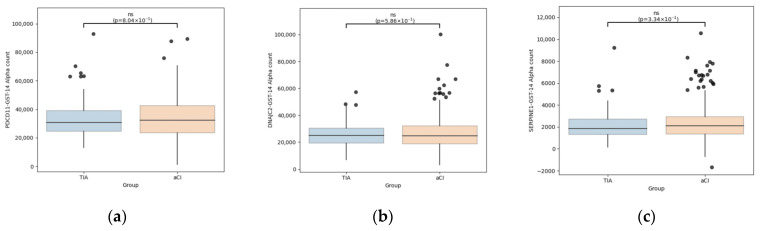
Comparison of antibody levels between acute ischemic stroke (AIS) and transient ischemic attack (TIA). Boxplots illustrate the distributions of (**a**) PDCD11-Ab, (**b**) DNAJC2-Ab, and (**c**) SERPINE1-Ab in the aCI and TIA groups. No statistically significant differences were observed between groups for any antibody marker, supporting the analytical approach of combining these ischemic subtypes into a single patient cohort.

**Figure A2:**
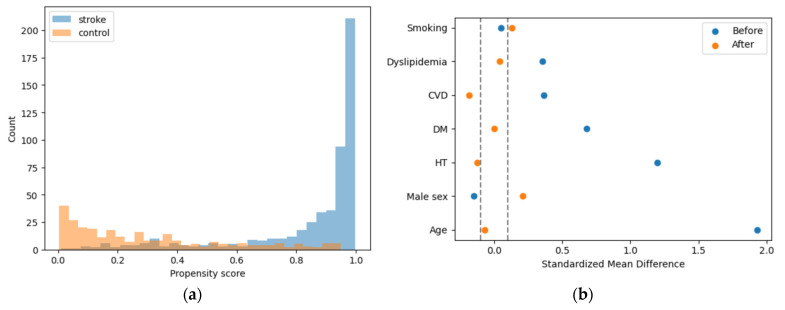
Propensity score matching and covariate balance. (**a**) Distribution of propensity scores in the unmatched cohort. Stroke patients showed substantially higher propensity scores compared with controls prior to matching. (**b**) Standardized mean differences (SMDs) for baseline covariates before and after 1:1 propensity score matching. Matching was performed using age, sex, hypertension, diabetes mellitus, cardiovascular disease, dyslipidemia, and smoking status. After matching, covariate imbalance was markedly reduced, with all absolute SMD values < 0.25, indicating improved balance between stroke and control groups.

Prespecified sensitivity analyses, including subtype comparison between atherothrombotic cerebral infarction and transient ischemic attack and propensity score matching, were performed to evaluate the robustness of the primary findings. The results were consistent with the main analyses and are provided in detail in Appendix A (Table A1 and Table A2 and Figure A1 and Figure A2).

## 3. Discussion

### 3.1. Central Finding: Identification of a Latent Antibody Axis

The present study identifies a latent antibody axis associated with vascular vulnerability in ischemic stroke through integrated statistical and machine-learning analyses. Several clinically and biologically relevant insights emerge from these findings.

A central finding of this study is the presence of a latent antibody axis associated with stroke vulnerability. Principal component analysis showed that the first component alone explained approximately 79% of the variance, strongly suggesting the presence of a latent antibody axis rather than independent marker effects. Furthermore, this principal component was significantly associated with stroke (OR 1.62) and remained independently predictive after adjustment for age, hypertension, and diabetes mellitus (OR 1.25), despite showing only a modest correlation with aging (r = 0.30).

This distinction is clinically meaningful because it suggests that the antibody signal is unlikely to represent a mere consequence of immunosenescence [12]. Instead, elevated antibody levels may reflect biological vulnerability beyond chronological aging, potentially capturing cumulative vascular injury, immune dysregulation, or other pathophysiological processes relevant to stroke development, consistent with emerging frameworks emphasizing integrated biological signals in complex disease [5].

Unsupervised clustering further reinforced this interpretation by identifying two biologically distinct subgroups with markedly different stroke prevalence (82% vs. 59%). The higher-risk cluster was characterized by consistently elevated levels across all three antibodies, suggesting that overall antibody burden may represent cumulative vascular or immunological stress rather than isolated pathological pathways.

Collectively, these findings support the presence of a latent antibody axis associated with vascular vulnerability. From this perspective, antibody profiling may help illuminate interindividual heterogeneity that is not fully explained by conventional risk factors alone, aligning with emerging precision medicine approaches aimed at biologically informed risk stratification [13].

### 3.2. Biological Interpretation of the Antibody Signature

The three antibody markers evaluated in this study—PDCD11-Ab, DNAJC2-Ab, and SERPINE1-Ab—are biologically plausible candidates for reflecting vascular injury and immune activation, as each has mechanistic links to pathways implicated in ischemic stroke.

PDCD11 regulates NF-κB-dependent Fas ligand (FasL) expression [14], and Fas–FasL signaling has been shown to contribute to apoptotic and inflammatory responses in experimental stroke models [15]. The accumulation of PDCD11-positive cells within ischemic lesions and the elevation of circulating anti-PDCD11 antibodies in acute ischemic stroke further support its association with inflammation-driven cellular injury [8].

DNAJC2, a cochaperone belonging to the Hsp40/DnaJ family, is involved in cellular stress responses and protein quality control. Hsp40 family proteins are upregulated in unstable atherosclerotic plaques [16], suggesting a role in plaque vulnerability and vascular remodeling. In addition, elevated anti-DNAJC2 antibodies have been reported in patients with atherosclerotic diseases, including ischemic stroke [6], supporting the hypothesis that DNAJC2-related immune responses may reflect chronic vascular stress and proteostatic imbalance.

SERPINE1 encodes plasminogen activator inhibitor-1 (PAI-1), a central regulator of fibrinolysis. Increased PAI-1 enhances thrombogenic potential and vascular inflammation [17], and elevated circulating PAI-1 has been consistently associated with cardiovascular risk [18]. The presence of anti-PAI-1 (SERPINE1) antibodies in ischemic stroke further suggests a link between impaired fibrinolytic regulation and humoral immune activation [7].

Although these proteins are linked to distinct biological pathways—including apoptosis, cellular stress, and thrombosis—our findings suggest that they may converge on a shared underlying process. Rather than functioning as isolated predictors, their combined expression appears to capture an integrated biological state, consistent with prior evidence that multimarker strategies better reflect the complexity of vascular disease [3].

One plausible interpretation is that elevated antibody levels reflect cumulative vascular injury rather than single pathological events, consistent with established models of chronic vascular inflammation and endothelial injury [4]. Chronic endothelial damage, inflammatory activation, and repeated subclinical insults may promote sustained antigen exposure, thereby triggering humoral immune responses detectable as circulating autoantibodies, a phenomenon aligned with chronic low-grade inflammatory processes described in vascular aging [12].

Together, these findings support a model in which autoantibodies function not merely as disease markers but as indicators of cumulative biological stress.

### 3.3. Nonlinear Contributions Revealed by SHAP

Model interpretability analyses provided critical context for these observations. SHAP identified antibody markers as influential predictors, ranking immediately after the major clinical risk factors—age, hypertension, and diabetes mellitus. This positioning indicates that antibody markers contribute meaningfully to risk estimation despite limited changes in global performance metrics.

Interestingly, SHAP dependence plots demonstrated heterogeneous and non-monotonic effects across antibody levels, suggesting nonlinear relationships with stroke risk that may not be adequately captured by conventional linear models, consistent with prior work highlighting the ability of interpretable machine-learning methods to reveal complex feature interactions [19,20]. These nonlinear patterns are compatible with threshold-dependent biological processes, in which risk accelerates once cumulative stress surpasses compensatory capacity.

The convergence of nonlinear SHAP effects, principal component structure, and clustering patterns strongly indicates that combined antibody signatures capture an integrated biological signal linked to subclinical vascular injury.

### 3.4. Independent Association of DNAJC2 Antibody

Importantly, multivariable logistic regression identified DNAJC2-Ab as an independent predictor of stroke even after adjustment for major clinical risk factors, whereas PDCD11-Ab and SERPINE1-Ab were not statistically significant individually. This pattern suggests that although individual antibodies may show variable statistical strength, antibody-related immune responses nonetheless capture clinically meaningful biological information, consistent with extensive evidence linking immune activation and vascular disease [4].

Furthermore, in propensity score-matched analyses (120 matched pairs), DNAJC2-Ab retained its independent association despite reduced overall predictive performance in the matched cohort. The consistency of this association across conventional regression, machine-learning, and matched analyses strengthens the inference that DNAJC2-related immune activation reflects a robust biological signal rather than residual confounding.

### 3.5. Predictive Performance in the Context of High Baseline Discrimination

Models based solely on clinical variables demonstrated excellent discrimination, with bootstrap AUCs of 0.916 for Random Forest and 0.919 for logistic regression. These results underscore the dominant predictive contribution of established vascular risk factors such as age, hypertension, and diabetes mellitus. When baseline performance already exceeds an AUC of 0.90, achieving substantial improvements in global metrics becomes inherently challenging because much of the explainable risk is already captured by conventional variables [21]. This ceiling effect is particularly relevant in high-performing models, where even biologically meaningful signals may not translate into large changes in AUC.

Notably, the addition of antibody markers resulted in highly similar performance (AUC 0.913 for Random Forest and 0.923 for logistic regression), with overlapping confidence intervals, and did not meaningfully improve risk reclassification. Net reclassification improvement (NRI) and integrated discrimination improvement (IDI) analyses showed no evidence of incremental benefit and were numerically negative. At first glance, these findings might suggest limited incremental value. However, such an interpretation would overlook the biological insights revealed by subsequent analyses. The absence of meaningful improvement in reclassification is not unexpected and aligns with established principles for evaluating the incremental value of new biomarkers, particularly in models with already high baseline discrimination [22].

Importantly, calibration remained preserved, as reflected by modest Brier scores. This suggests that the incorporation of antibody information did not compromise model reliability, even if improvements in AUC were limited.

### 3.6. Consistency Across Modeling Strategies

The overall consistency between conventional regression and machine-learning models strengthens the validity of the findings and reduces the likelihood that the observed associations are artifacts of a single modeling strategy. Subtype sensitivity analysis further demonstrated no significant differences in antibody levels between atherothrombotic cerebral infarction and transient ischemic attack, supporting the validity of combining these entities into a unified ischemic cohort. This suggests that the antibody-related signal reflects broader vascular vulnerability rather than subtype-specific pathology.

### 3.7. Limitations and Future Directions

Several limitations should be acknowledged. First, the principal component and clustering analyses were exploratory and should be interpreted cautiously. Second, the cross-sectional design precludes causal inference regarding whether these antibodies contribute directly to stroke pathogenesis or represent downstream epiphenomena. Third, the study population exhibited a substantial age difference between stroke patients and healthy controls, which represents an inherent limitation of this case–control design. Because ischemic stroke predominantly affects older individuals, recruiting truly healthy age-matched controls is challenging in real-world settings. Although age was rigorously adjusted for in multivariable models and propensity score matching analyses, residual confounding related to age cannot be fully excluded.

Importantly, SHAP analysis confirmed that age was the dominant predictor in the supervised models, highlighting the strong influence of established clinical risk factors in case–control machine-learning settings. This observation also underscores a broader methodological limitation: when major risk factors such as age already provide high discriminative power, supervised learning approaches may have limited ability to detect incremental contributions from novel biomarkers in global performance metrics. Notably, however, the principal antibody component showed only a modest correlation with age and remained independently associated with stroke after adjustment, suggesting that the identified antibody axis may capture biological processes beyond chronological aging alone.

Future prospective cohort studies, particularly those enriched for younger or intermediate-risk populations, will be important to further clarify the age-independent role of the antibody-related signal and to determine its potential value for early risk stratification. Additionally, the study population was derived from a single cohort, which may limit broader applicability. Finally, external validation in independent cohorts will be necessary to confirm generalizability.

## 4. Materials and Methods

### 4.1. Study Population

A total of 834 serum samples were initially collected, including 285 healthy controls and 549 patients with acute ischemic stroke (AIS) or transient ischemic attack (TIA). Healthy controls showed no evidence of ischemic lesions on magnetic resonance imaging.

One sample demonstrated an extreme outlier value for the SERPINE1-Ab that was considered likely attributable to measurement error. This sample was excluded prior to analysis to avoid distortion of statistical estimates, resulting in a final eligible cohort of 833 participants.

Clinical variables included age, sex, hypertension, diabetes mellitus, dyslipidemia, cardiovascular disease, and smoking status. Smoking was incorporated as an additional clinical covariate; however, smoking data were unavailable for six participants. Therefore, analyses requiring this variable—including multivariable regression and machine-learning models—were conducted in 827 individuals using a complete-case approach.

Patients with known autoimmune disorders were excluded to minimize potential confounding effects on autoantibody levels.

In the primary analyses, patients with AIS and TIA were combined into a single ischemic cohort. This decision was supported by sensitivity analyses demonstrating no significant differences in antibody levels between the two subtypes.

### 4.2. Measurement of Serum Autoantibodies

Serum autoantibodies against PDCD11-Ab, DNAJC2-Ab, and SERPINE1-Ab were quantified using amplified luminescence proximity homogeneous assay-linked immunosorbent assay (AlphaLISA) [23] on 384-well white opaque microtiter plates (OptiPlate™, Revvity, Waltham, MA, USA). Recombinant antigens were expressed and purified as previously described [6,7,8].

Serum samples were diluted 1:100, incubated with glutathione-conjugated donor beads, and reacted with anti-human IgG-conjugated acceptor beads. Alpha counts were measured on day 14 according to the standardized laboratory protocol. This time point was selected because signal intensity stabilizes following sufficient antigen–antibody complex formation, thereby improving assay reproducibility and minimizing variability related to incomplete signal development.

To correct for nonspecific binding, GST-derived signals were subtracted from antigen-bead signals, and GST-corrected AlphaLISA values were used for all analyses. All assays were performed blinded to clinical status.

### 4.3. Statistical Analysis

#### 4.3.1. Conventional Statistical Analysis

All statistical analyses were conducted using Python (version 3.10.9).

Continuous variables were compared using the Mann–Whitney U test, and categorical variables were analyzed using the chi-squared test. Multivariable logistic regression was performed to evaluate independent associations between antibody markers and ischemic stroke after adjusting for major clinical risk factors, including age, sex, hypertension, diabetes mellitus, dyslipidemia, cardiovascular disease, and smoking status. Antibody variables were standardized (mean = 0, SD = 1).

Odds ratios (ORs) and 95% confidence intervals (CIs) were calculated. Multicollinearity was assessed using variance inflation factors.

Model discrimination was assessed using receiver operating characteristic (ROC) curves and the area under the curve (AUC). Ninety-five percent confidence intervals (95% CIs) were calculated where appropriate. A two-sided *p*-value < 0.05 was considered statistically significant.

Missing data were handled using complete-case analysis. Participants were excluded only from analyses requiring the missing variables. For machine-learning analyses, complete-case data (n = 827) were used to ensure consistency across modeling approaches.

#### 4.3.2. Machine-Learning Models

Machine-learning models were employed to complement conventional regression analyses and to explore potential nonlinear associations between antibody markers and stroke risk.

Two algorithms were implemented using scikit-learn:

- Logistic Regression
- Random Forest [10]

For each algorithm, two models were constructed:

1. Clinical-only model
2. Clinical + antibody marker model

The dataset was randomly divided into training and test sets, with 25% of the data reserved for testing using stratified sampling with a fixed random seed (random_state = 0). Model development was conducted exclusively within the training set, and final model performance was evaluated using the independent test dataset. A fixed random seed was used to ensure reproducibility.

Complete-case data (n = 827) were used for machine-learning analyses.

#### 4.3.3. Hyperparameter Tuning

Hyperparameter tuning for both logistic regression and random forest models was performed within the training dataset using GridSearchCV with the area under the receiver operating characteristic curve (AUC) as the scoring metric and cross-validation.

For logistic regression, the search space included the inverse regularization strength (C = 0.01–10) and class weighting (none or balanced), while the penalty (L2) and solver (lbfgs) were fixed to ensure numerical stability.

For the random forest model, the search space included the number of trees (200–500), maximum depth (3–6 or unlimited), minimum samples required for splitting (2 or 10), minimum samples per leaf (1–5), feature subsampling strategy (sqrt or log2), and class weighting (none or balanced).

The best-performing hyperparameters were selected based on cross-validated AUC, and the final models were refitted on the full training dataset. Hyperparameter tuning was conducted exclusively within the training data to prevent information leakage. Detailed tuning settings and selected parameters are provided in the Appendix A.

#### 4.3.4. Model Evaluation

Discriminative performance was assessed using receiver operating characteristic (ROC) curves and AUC. To quantify uncertainty in model performance, 95% confidence intervals were estimated using bootstrap resampling.

Model calibration was evaluated using the Brier score [24], a measure of agreement between predicted probabilities and observed outcomes.

To determine whether the addition of antibody markers improved predictive performance beyond AUC comparisons, net reclassification improvement (NRI) and integrated discrimination improvement (IDI) were calculated [22]. Category-free NRI was calculated.

#### 4.3.5. Model Interpretability

To enhance the interpretability of the machine-learning models, SHapley Additive exPlanations (SHAP) were used to quantify feature contributions and identify influential predictors [19]. Dependence plots were generated to explore potential nonlinear relationships between antibody markers and stroke risk. SHAP interaction values were also calculated to evaluate potential non-additive relationships between antibody markers and clinical variables.

#### 4.3.6. Exploratory Analyses

Exploratory analyses were conducted to investigate latent biological structures underlying antibody responses.

Principal component analysis (PCA) was performed using standardized antibody levels to evaluate shared variance among markers [25]. The first principal component (PC1) was retained as a summary measure of overall antibody burden.

Subsequently, unsupervised k-means clustering was applied to standardized antibody levels to identify potential biologically distinct subgroups [26]. The optimal number of clusters was determined using silhouette analysis [27]. Because these analyses were hypothesis-generating, results were interpreted cautiously.

Sensitivity analyses, including subtype comparison and propensity score matching (PSM), were prespecified to assess the robustness of the primary findings. Detailed procedures and results are provided in Appendix A.

#### 4.3.7. Sensitivity Analysis

Sensitivity analyses, including subtype comparison and propensity score matching (PSM) [28], were conducted to assess the robustness of the primary findings. Detailed procedures and results are described in Appendix A.

## 5. Conclusions

This study identified a latent antibody axis associated with vascular vulnerability in ischemic stroke through integrated statistical and machine-learning analyses. Principal component and clustering approaches consistently revealed biologically distinct high-risk phenotypes characterized by elevated overall antibody burden.

These findings suggest that the combined antibody signature reflects cumulative vascular and immunological stress rather than isolated pathological pathways. The heterogeneous and nonlinear contributions of these antibodies—captured through interpretable machine-learning methods—highlight the potential value of biologically informed risk characterization beyond conventional factor-based prediction.

Notably, integration of PDCD11, DNAJC2, and SERPINE1 antibody levels with established clinical risk factors did not result in meaningful improvement in global predictive performance, likely reflecting the already high discriminative power of traditional risk factors.

Although immediate clinical implementation is premature, a multimarker antibody framework supported by machine-learning methodologies may improve understanding of stroke-related biological vulnerability and contribute to future precision prevention strategies. Prospective validation in independent cohorts will be essential prior to clinical translation.

## Figures and Tables

**Table 1:** Comparison of clinical characteristics between stroke patients and controls.

	Patient (*n* = 543)	Healthy (*n* = 284)
Age	77.0 (68.0–84.0) *	54.0 (43.8–60.0)
Male sex	316 (58.2%)	186 (65.5%)
Hypertension	387 (71.3%) *	57 (20.1%)
Diabetes mellitus	148 (27.3%) *	11 (3.9%)
Dyslipidemia	154 (28.4%) *	40 (14.1%)
Cardiovascular disease	44 (8.1%) *	2 (0.7%)

Continuous variables are presented as median (interquartile range) and were compared using the Mann–Whitney U test. Categorical variables are presented as counts (percentages) and were compared using the chi-square test. * *p* < 0.05 was considered statistically significant.

**Table 2:** Multivariable logistic regression analysis identifying factors associated with ischemic stroke.

	Odds Ratio	95% CI	*p*-Value
Age	1.14	1.12–1.16	<0.001 *
Male sex	1.15	0.67–1.97	0.604
Hypertension	3.17	2.00–5.02	<0.001 *
Diabetes mellitus	6.47	2.90–14.45	<0.001 *
Dyslipidemia	1.00	0.58–1.73	0.998
Cardiovascular disease	0.95	0.20–4.40	0.944
Smoking	1.14	0.68–1.89	0.624
PDCD11-Ab	0.94	0.61–1.46	0.791
DNAJC2-Ab	1.83	1.18–2.83	0.007 *
SERPINE1-Ab	0.77	0.56–1.07	0.125

Odds ratios were estimated using multivariable logistic regression, adjusting for all listed covariates. Continuous antibody variables were standardized (mean = 0, SD = 1), and odds ratios represent the change in odds per one standard deviation increase. Binary variables were coded as 0 (absence) and 1 (presence). Model fit: McFadden pseudo-R^2^ = 0.51; likelihood ratio test *p* < 0.001. No evidence of multicollinearity was observed (all variance inflation factors < 5). * *p* < 0.05 was considered statistically significant.

**Table 3:** Reclassification metrics for machine-learning models with the addition of antibody markers.

Model	Metric	Estimate	95% CI
Random Forest	NRI (overall)	−0.81	−1.06 to −0.54
Random Forest	NRI (events)	−0.46	−0.61 to −0.31
Random Forest	NRI (nonevents)	−0.35	−0.56 to −0.13
Random Forest	IDI	−0.058	−0.080 to −0.037
Logistic Regression	NRI (overall)	−0.74	−1.00 to −0.48
Logistic Regression	NRI (events)	−0.53	−0.66 to −0.38
Logistic Regression	NRI (nonevents)	−0.21	−0.43 to 0.02
Logistic Regression	IDI	−0.023	−0.040 to −0.005

NRI, net reclassification improvement; IDI, integrated discrimination improvement; CI, confidence interval.

## Data Availability

The data that support the findings of this study are available from the corresponding author upon reasonable request. The data are not publicly available due to privacy and ethical restrictions.

## Abbreviations

The following abbreviations are used in this manuscript:

Ab	Antibody
AIS	Acute Ischemic Stroke
AUC	Area Under the Curve
CI	Confidence Interval
CV	Cross-Validation
DM	Diabetes Mellitus
HT	Hypertension
IDI	Integrated Discrimination Improvement
IQR	Interquartile Range
LR	Logistic Regression
ML	Machine Learning
NRI	Net Reclassification Improvement
OR	Odds Ratio
PCA	Principal Component Analysis
PC1	First Principal Component
PSM	Propensity Score Matching
RF	Random Forest
ROC	Receiver Operating Characteristic
SHAP	SHapley Additive exPlanations
SMD	Standardized Mean Difference
TIA	Transient Ischemic Attack

## Appendix A

### Appendix A.1. Machine-Learning Model Development and Hyperparameter Optimization

#### Appendix A.1.1. Cross-Validation and Data Splitting

Machine-learning analyses were performed using the complete-case dataset (n = 827). The data were divided into training (75%) and test (25%) sets using stratified sampling with a fixed random seed (random_state = 0).

Within the training dataset, hyperparameter optimization was conducted using five-fold stratified cross-validation (StratifiedKFold, n_splits = 5, shuffle = True, random_state = 0) to prevent information leakage.

#### Appendix A.1.2. Hyperparameter Search Space

Hyperparameter tuning was performed using GridSearchCV with AUC as the scoring metric.

Logistic Regression

- C: 0.01–10
- class_weight: None or balanced
- penalty: L2 (fixed)
- solver: lbfgs (fixed)

Random Forest

- n_estimators: 200–500
- max_depth: 3–6 or None
- min_samples_split: 2 or 10
- min_samples_leaf: 1–5
- max_features: sqrt or log2
- class_weight: None or balanced

#### Appendix A.1.3. Final Selected Hyperparameters

**Table A1:** Logistic Regression (Clinical + Antibody Model).

Parameter	Selected Value
C	0.1
Penalty	L2
Solver	lbfgs
class_weight	balanced

**Table A2:** Logistic Regression (Clinical-Only Model).

Parameter	Selected Value
C	1
Penalty	L2
Solver	lbfgs
class_weight	balanced

**Table A3:** Random Forest (Clinical + Antibody Model).

Parameter	Selected Value
n_estimators	500
max_depth	3
min_samples_split	2
min_samples_leaf	5
max_features	sqrt
class_weight	balanced

**Table A4:** Random Forest (Clinical-Only Model).

Parameter	Selected Value
n_estimators	200
max_depth	4
min_samples_split	10
min_samples_leaf	2
max_features	sqrt
class_weight	balanced

#### Appendix A.1.4. Model Refitting and Evaluation

Final models were refitted on the full training dataset and evaluated on the independent test dataset. Performance was assessed using AUC with bootstrap-derived 95% confidence intervals, Brier score for calibration, and NRI/IDI for incremental predictive value.

### Appendix A.2. Sensitivity Analyses

#### Appendix A.2.1. Subtype Comparison (AIS vs. TIA)

To assess the appropriateness of analyzing acute ischemic stroke (AIS) and transient ischemic attack (TIA) as a unified ischemic stroke group, a subtype comparison was performed (Table A1; Figure A1).

**Table A5:** Clinical characteristics of patients with acute ischemic stroke (AIS) and transient ischemic attack (TIA).

	AIS (*n* = 457)	TIA (*n* = 86)
Age	78.0 (68.0–84.0) *	73.0 (62.5–80.0)
Male sex	266 (58.2%)	50 (58.1%)
Hypertension	332 (73.7%)	55 (64.0%)
Diabetes mellitus	123 (26.9%)	25 (29%)
Dyslipidemia	120 (26.3%)	34 (39.5%)
Cardiovascular disease	39 (8.5%)	5 (5.81%)
Smoking	226 (49.4%)	40 (46.5%)

Baseline demographic and vascular risk factors were compared between the AIS (n = 457) and TIA (n = 86) groups. Age differed significantly between groups, whereas no significant differences were observed for sex, hypertension, diabetes mellitus, dyslipidemia, cardiovascular disease, smoking status, antibody levels, or principal component scores. * *p* < 0.05 was considered statistically significant.

Age differed significantly between subtypes; however, no statistically significant differences were observed in individual antibody levels between the two groups. These findings support the validity of combining AIS and TIA into a single patient cohort for the primary analyses and suggest that the observed antibody-related signal was not driven by a specific ischemic subtype.

#### Appendix A.2.2. Propensity Score Matching

Propensity score matching (PSM) was performed to further address potential confounding due to baseline imbalances (Figure A2; Table A2).

**Table A6:** Multivariable logistic regression analysis in the propensity score–matched cohort (1:1 matching).

Variable	OR	95% CI	*p*-Value
Age	1.14	1.12–1.16	<0.001 *
Male sex	1.23	0.76–1.97	0.402
Hypertension	3.18	2.01–5.04	<0.001 *
Diabetes mellitus	6.43	2.89–14.32	<0.001 *
Dyslipidemia	1.00	0.58–1.72	0.992
Cardiovascular disease	0.93	0.20–4.34	0.931
PDCD11-Ab	0.94	0.61–1.46	0.782
DNAJC2-Ab	1.85	1.20–2.86	0.005 *
SERPINE1-Ab	0.77	0.55–1.06	0.113

Antibody variables were standardized prior to analysis. DNAJC2-Ab remained independently associated with ischemic stroke after matching for age, sex, hypertension, diabetes mellitus, cardiovascular disease, lipidemia, and smoking status. * *p* < 0.05 was considered statistically significant.

Propensity scores were estimated using logistic regression including age, sex, hypertension, diabetes mellitus, cardiovascular disease, dyslipidemia, and smoking status. One-to-one nearest-neighbor matching without replacement was performed, resulting in 120 matched pairs.

Covariate balance before and after matching was assessed using standardized mean differences (SMDs), with values < 0.25 considered acceptable.

#### Appendix A.2.3. Analyses in the Matched Cohort

Regression and machine-learning analyses were repeated in the matched cohort to evaluate the robustness of the primary findings.

In the matched cohort, overall predictive performance decreased compared with the original cohort (AUC 0.56 for Random Forest; 0.61 for logistic regression), likely reflecting reduced sample size and attenuation of baseline confounding.

However, multivariable logistic regression yielded findings consistent with the primary analysis. In particular, DNAJC2-Ab remained independently associated with ischemic stroke, whereas PDCD11-Ab and SERPINE1-Ab were not statistically significant.
